# Bibliometric Analysis of Diagnostic Yield of CT Pulmonary Angiogram (CTPA) in the Diagnosis of Pulmonary Embolism (PE)

**DOI:** 10.7759/cureus.41979

**Published:** 2023-07-16

**Authors:** Ren Yi Kow, Khairiah Razali, Chooi Leng Low, Khairul Nizam Siron, Zamzuri Zakaria Mohamad, Mubarak Mohd Yusof, Ahmad Razali Md Ralib Md Raghib

**Affiliations:** 1 Orthopaedics, Traumatology and Rehabilitation, International Islamic University Malaysia, Kuantan, MYS; 2 Basic Medical Sciences, International Islamic University Malaysia, Kuantan, MYS; 3 Radiology, International Islamic University Malaysia, Kuantan, MYS; 4 Orthopaedics, Traumatology and Rehabilitation, International Islamic Univeristy, Kuantan, MYS; 5 Radiology, Hospital Tengku Ampuan Afzan, Kuantan, MYS

**Keywords:** ct pulmonary angiogram, radiology research, review article, bibliometric analyis, pulmonary emboli

## Abstract

CT pulmonary angiography (CTPA) is the investigation of choice for diagnosing pulmonary embolism (PE). Due to the speed and ease of performing the CTPA scans, more clinicians are becoming overly reliant on them, even for patients without strong suspicion of PE. We conducted a bibliometric analysis on the PubMed database from 1990 to 2022 to investigate the literature on the diagnostic yield of CTPA in the diagnosis of PE. A total of 166 articles were published in 98 journals. The number of publications has steadily increased since 2010 and peaked in 2020. Authors from 24 countries contributed to these publications, with the overwhelming majority emanating from United States of America, totaling 66 articles. The authors’ contributions were relatively well spread out, with the top four authors publishing the highest number of articles at six each. When we investigated the collaboration between the authors, we found limited multinational and multi-institutional collaborations on this topic. Therefore, more multinational and multi-institutional collaborations will be valuable in future studies. In conclusion, this bibliometric analysis summarizes the literature on diagnostic yield of CTPA in the diagnosis of PE and sheds light on the future pathway that researchers and institutions can focus on.

## Introduction and background

Pulmonary embolism (PE) is a life-threatening condition caused by blood clots in the pulmonary arteries [1-3]. Worldwide, PE is the third most common cause of cardiocascular death after stroke and heart attack [1,2]. PE has a mortality rate of up to 25% if it is left untreated [1]. With the advancement in medical imaging technology, CT pulmonary angiography (CTPA) has become the imaging of choice to diagnose PE. In addition to its swift scanning time, CTPA offers the advantage of high sensitivity and specificity. The sensitivity and specificity of CTPA in diagnosing PE range between 96% and 100% and between 89% and 98%, respectively [1-3]. Consequently, more clinicians are increasingly relying on CTPA to rule out PE [1-3].

However, despite these obvious advantages, CTPAs are not without their own risks. They expose patients to a median radiation dose of 10 mSV, which is equivalent to 137 plain radiographs of the chest [4]. This radiation poses a risk of stochastic radiation-induced malignancy to patients. Furthermore, CTPAs may also cause complications resulting from intravenous contrast administration, such as contrast-induced nephropathy [1-4]. With the increasing number of CTPAs being performed worldwide, clinicians are becoming more aware of the potential complications associated with CTPAs.

This review aims to conduct a bibliometric analysis of the literature on the diagnostic yield of CTPA in the diagnosis of PE. Bibliometric analysis is a great way to assess the scholarly impact of scientific publications [5,6]. It helps summarize the research interest, identify emerging trends, and reveal collaboration patterns in the published literature [5,6]. Additionally, it serves as a valuable tool to identify under-explored areas of research topic, providing useful insights and guidance to the researchers and institutions [5,6].

## Review

Literature search and search strategy

The bibliometric data was extracted from the PubMed advanced search engine. A review by Falagas et al. compared the differences between PubMed and other medical databases, such as Scopus, Web of Science, and Google Scholar [7]. They found that PubMed is a user-friendly medical database that is handy and easy to use. PubMed was the most frequently used database for information in the biomedical field due to its ease of use and free availability. PubMed is widely recognized for its reliability in providing datasets, which, in turn, facilitates bibliometric analysis and tracking of essential information such as author names, keywords, affiliations, countries, journal titles, and broad subject areas.

**Figure 1:**
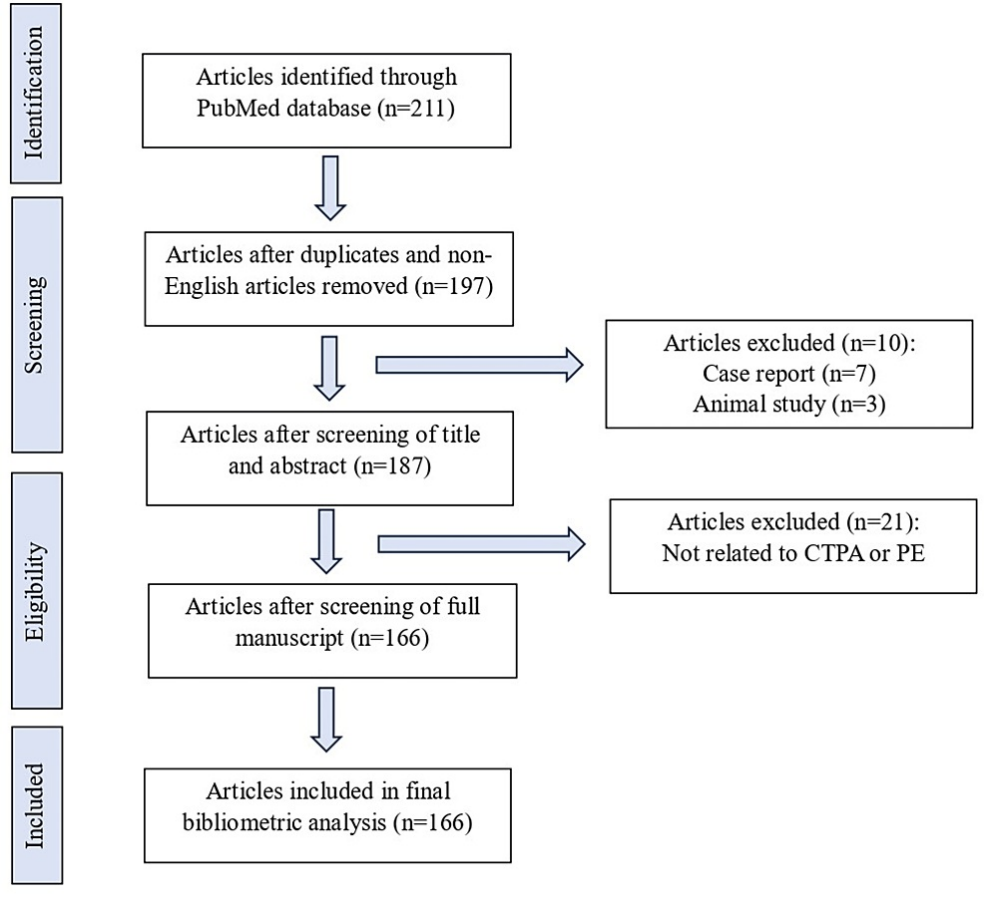
Flowchart of search results CTPA: CT pulmonary angiography; PE: Pulmonary embolism

The Pubmed database was searched from 1990 to 2022, using the following keywords: “(CTPA OR computed tomography pulmonary angiogram) AND (pulmonary embol*) AND (yield)”. The data was analyzed using R Studio 2021 for Windows with the “bibliometrix” package installed in R and Microsoft Excel 2019 (Microsoft ® Corp., Redmond, WA). Information such as document type, keywords, authors, country, sources, and other essential parameters were extracted from the PubMed database using the R software. The articles were screened by the authors for suitability (Figure 1). The presentation of data, including illustrative graphs, was completed using “bibliometrix” and Microsoft Excel 2019 [5,6,8].

Results

Annual Scientific Production

**Figure 2:**
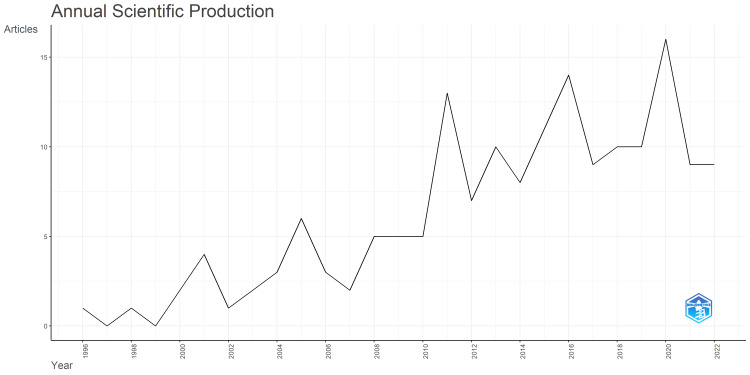
A graphical representation of the annual scientific production from 1990 to 2022

A total of 211 articles were retrieved from the PubMed database from the year 1990 to 2022, using the search terms presented (Figure 1). After screening by the authors, only 166 articles were analyzed in this review (Figure 1). As shown in Figure 2, the first related article was published in 1996, and the number of annual publications remained low until 2010, when the annual publication rate started to increase steadily. The annual scientific production on this topic reached its peak in 2020, with 16 publications during that year. These trends suggest a shift of focus toward the impact of radiation posed by CTPA and the aim to reduce it.

Journals and Number of Publications

**Figure 3:**
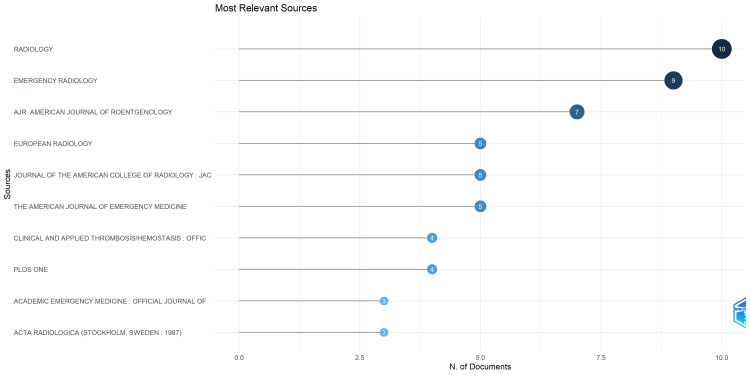
A graphical representation of the number of publications in journals

During the study period, the 166 articles included in this review were published across 98 journals. As demonstrated in Figure 3, the journal Radiology had the most publications, with a total of 10 articles published in that journal. This was closely followed by Emergency Radiology (nine articles) and American Journal of Roentgenology (AJR; seven articles). The European Radiology, Journal of The American College of Radiology: JACR, and The American Journal of Emergency Medicine had five publications each. Clinical and Applied Thrombosis/Hemostasis and PLOS ONE had four publications each, while Academic Emergency Medicine and Acta Radiologica had three publications each.

Countries and Their Publication Number

**Figure 4:**
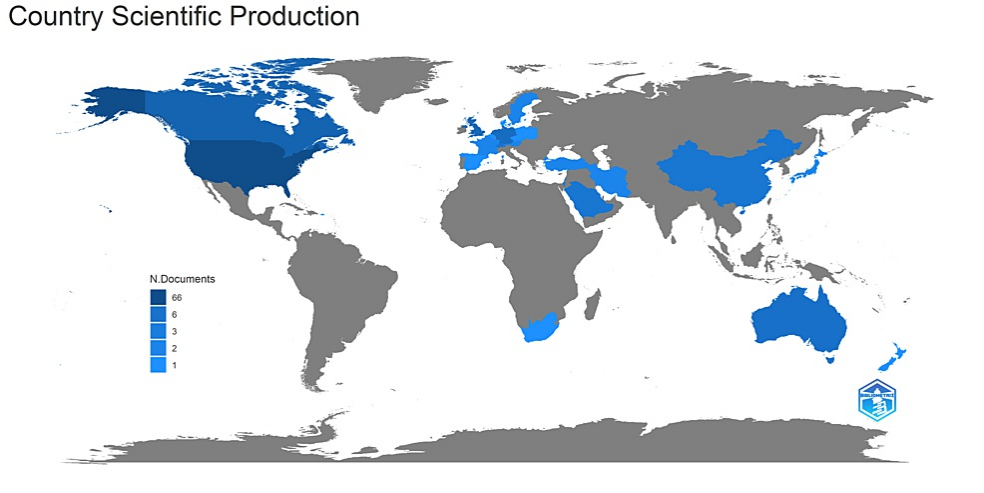
A graphical illustration of the country-wise scientific production on the world map

The articles were produced by authors from 24 countries. The overwhelming majority of the articles were published by authors from United States of America, with a total publication of 66 articles (Table 1). This was followed by Canada (16 articles), United Kingdom (12 articles), Germany and Netherlands (each with nine articles), Australia (seven articles), China, Saudi Arabia and Switzerland (each with five articles). When investigating the distribution of articles on the world map, it became evident that article production was still lacking in many parts of the world (Figure 4).

**Table 1:** Table highlighting the country-wise scientific production Only countries with five or more publications are highlighted in this table.

Country	Number of publications
United States of America	66
Canada	16
United Kingdom	12
Germany	9
Netherlands	9
Australia	7
China	5
Saudi Arabia	5
Switzerland	5

Authors and Their Publication Number

**Figure 5:**
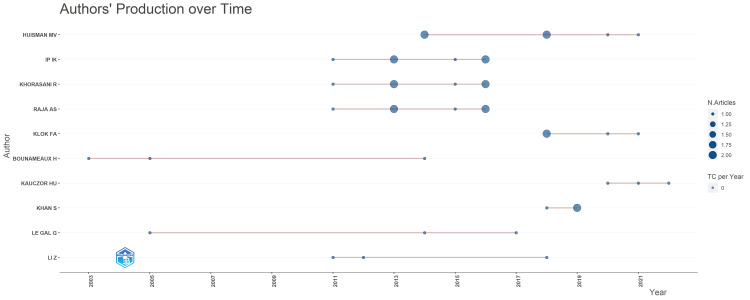
A graphical representation of the annual production analysis of the top authors over time

A total of 911 authors contributed to the production of these articles in this review. The contribution was relatively well spread out, with the highest number of publications at six articles. Huisman MV, Ip IK, Khorasani R, and Raja AS each contributed six articles in this review (Table 2). This was followed by Klok FA (four articles), Bounameaux H, Kauczor HU, Khan S, Le Gal G and Li Z (each contributed three articles). It is also worth noting that all top five contributors with the highest number of publications started publishing their research after the year 2010 (Figure 5).

**Table 2:** Table highlighting the top authors' scientific contribution

Author	Number of publications
Huisman MV	6
Ip IK	6
Khorasani R	6
Raja AS	6
Klok FA	4
Bounameaux H	3
Kauczor Hu	3
Khan S	3
Le Gal G	3
Li Z	3

Collaboration between Authors

**Figure 6:**
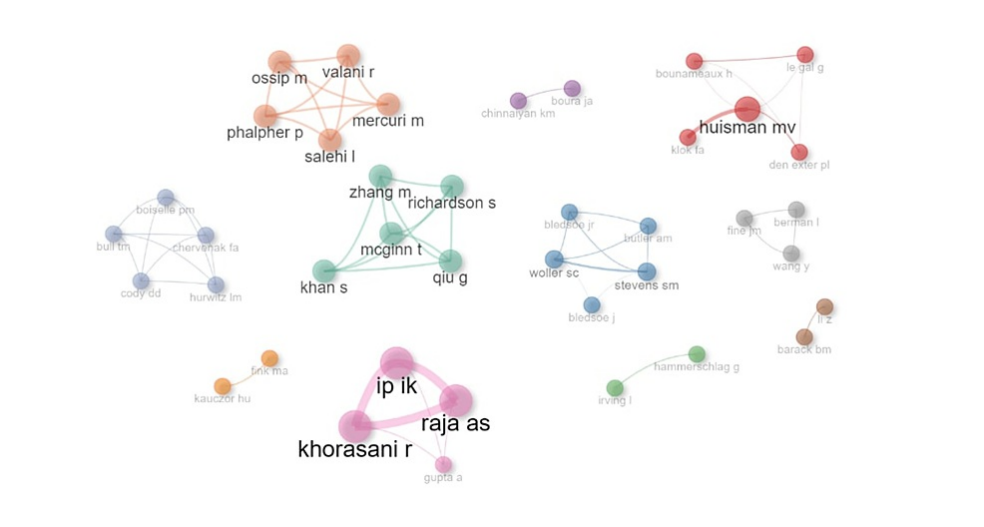
A graphical representation of the authors' collaboration network

**Figure 7:**
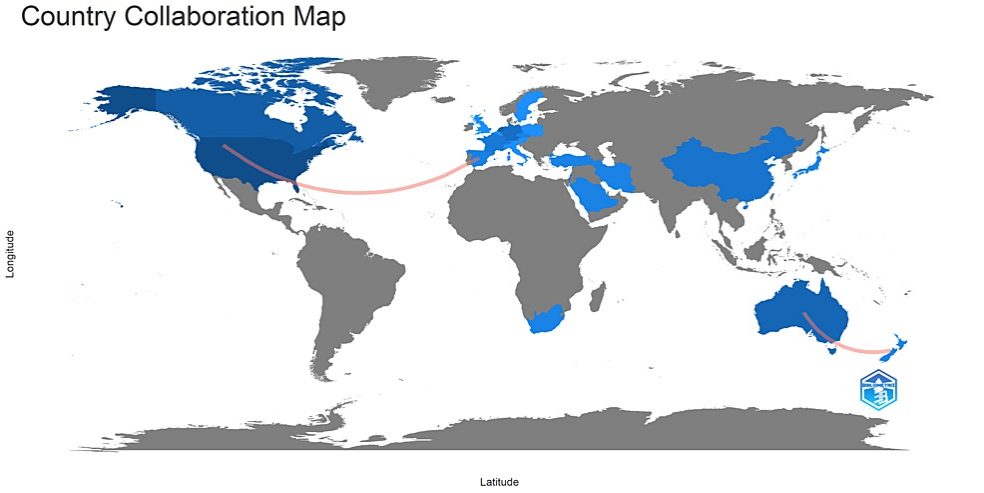
A graphical representation of the collaborations between different countries. The pink line depict the collaboration between different countries.

When we investigated the collaboration between the authors, we found limited multinational and multi-institutional collaborations. This is clearly illustrated in Figure 6, where there were minimal links between different authors. The same trend was observed in Figure 7, with few collaborations between different countries. The only partnerships observed between countries were between Australia and New Zealand, as well as between United States of America and European countries.

Discussion

In United States of America, up to 2.4 million of CTPA scans are carried out yearly to diagnose PE in the emergency department [1,2]. Due to the speed and ease of performing the CTPA scans, more clinicians are becoming overly reliant on them, even for patients without strong suspicion of PE. Nevertheless, unjustified CTPAs needlessly expose patients to radiation. For each CTPA performed, the patient is exposed to a lifetime malignancy risk of 2.8% and up to 14% risk of contrast-induced nephropathy [1-3]. As clinicians become more aware of the risk poses by the CTPAs, more studies are being published on the yield of CTPA in the diagnosis of PE. To our knowledge, there has been no previous bibliometric analysis on the diagnostic yield of CTPA in the diagnosis of PE.

The number of publications in this field started to increase after the 2010 and it reached a peak of 16 publications in 2020. The peak coincided with the COVID-19 pandemic during the year 2020, hence many studies published in 2020 were COVID-19 related [9-12]. For example, Whyte et al. published an article entitled “Pulmonary embolism in hospitalized patients with COVID-19” in the journal Thrombosis Research [13]. In their study, they investigated the incidence of PE in hospitalized patients with COVID-19 and the diagnostic yield of CTPA for PE. 

In this bibliometric analysis, we noticed minimal multicenter and multi-institution collaboration. One of the few exceptions is a multi-institutional observational study by Mountain et al. [14]. Their research, published in PLoS One, was a retrospective study involving 15 emergency departments in Australia. 

Limitations

There are several limitations to this bibliometric analysis. First, the literature search was limited to PubMed database, and non-English papers were excluded. This exclusion of publications from databases such as Scopus and Web of Science, as well as non-English articles, may results in publishing bias. Second, we did not delve into the citation analysis of the published articles, as our aim was to investigate the publication trends in this field. Besides that, we did not compare the yield rates of all the publications in this bibliometric analysis, as some of the studies focus on a small cluster of patients, such as pregnant patients or patients with cystic fibrosis [15,16]. Despite these limitations, this bibliometric analysis identifies the shortcomings in this field, which may prompt researchers and clinicians to collaborate more on this topic.

## Conclusions

By analyzing papers published from 1990 to 2022, this bibliometric analysis provides an overview of available research on the diagnostic yield of CTPA in the diagnosis of PE. Since 2010, the number of publications related to this topic has increased, indicating the emphasis placed on avoiding unnecessary radiation exposure for patients. Nevertheless, our analysis demonstrates a limited multinational and multi-institutional collaboration in this field. Therefore, more researchers and clinicians are needed to collaborate on this topic.

## References

[1] Low C, Kow R, Abd Aziz A, Yusof MM, Bee CL, Kamarudin NA, Raghib AR (2023). Diagnostic yield of CT pulmonary angiogram in the diagnosis of pulmonary embolism and its predictive factors. Cureus.

[2] Zantonelli G, Cozzi D, Bindi A (2022). Acute pulmonary embolism: prognostic role of computed tomography pulmonary angiography (CTPA). Tomography.

[3] Schissler AJ, Rozenshtein A, Kulon ME (2013). CT pulmonary angiography: increasingly diagnosing less severe pulmonary emboli. PLoS One.

[4] Richardson S, Lucas E, Cohen SL, Zhang M, Qiu G, Khan S, McGinn T (2020). Predictors of overtesting in pulmonary embolism diagnosis. Acad Radiol.

[5] Hussain T, Corraes A, Walizada K (2022). HIV dementia: a bibliometric analysis and brief review of the top 100 cited articles. Cureus.

[6] Pandey P, Chakole S, Wanjari MB, Prasad R (2023). A bibliometric analysis of scientific research publications related to pesticide poisoning in the South Asian countries. Cureus.

[7] Falagas ME, Pitsouni EI, Malietzis GA, Pappas G (2008). Comparison of PubMed, Scopus, Web of Science, and Google Scholar: strengths and weaknesses. FASEB J.

[8] Bibliometrix command in R Program. Bibliometrix - Home. [ Jan; 2023 ]. 2023 (2023). Bibliometrix: an r-tool for comprehensive science mapping analysis. https://www.bibliometrix.org/home/.

[9] Kow RY, Khalid KA, Zakaria Z, Awang MS (2022). COVID-19 pandemic: two-year experience and response of a teaching hospital in Malaysia and the effect on postgraduate orthopaedic training. Malays Orthop J.

[10] Kow RY, Mohamad Rafiai N, Ahmad Alwi AA, Low CL, Ahmad MW, Zakaria Z, Zulkifly AH (2022). COVID-19 infodemiology: association between Google search and vaccination in Malaysian population. Cureus.

[11] Anwarali Khan MH, Kow RY, Ramalingam S, Ho JP, Jaya Raj J, Ganthel Annamalai K, Low CL (2021). COVID-19 collateral damage: management of periprosthetic joint infection in Malaysia. Cureus.

[12] Anwarali Khan M, Kow R, Ramalingam S (2023). Outcomes of geriatric hip fractures in a tertiary referral center in Malaysia during the COVID-19 pandemic. Cureus.

[13] Whyte MB, Kelly PA, Gonzalez E, Arya R, Roberts LN (2020). Pulmonary embolism in hospitalised patients with COVID-19. Thromb Res.

[14] Mountain D, Keijzers G, Chu K (2016). RESPECT-ED: rates of pulmonary emboli (PE) and sub-segmental PE with modern computed tomographic pulmonary angiograms in emergency departments: a multi-center observational study finds significant yield variation, uncorrelated with use or small PE rates. PLoS One.

[15] Sheen JJ, Haramati LB, Natenzon A (2018). Performance of low-dose perfusion scintigraphy and CT pulmonary angiography for pulmonary embolism in pregnancy. Chest.

[16] Mahan KS, Ahmad H, Keenan AG, Prekker ME, Kempainen RR (2022). Yield of chest computed tomography angiogram in cystic fibrosis patients with suspected pulmonary embolism. Clin Respir J.

